# Notes and News

**Published:** 1899-02-04

**Authors:** 


### Notes and News.

(Collected and Communicated.)

HOSPITAL MEETINGS.

Central London Ophthalmic Hospital, Gray's Inn Road, W.C.—Annual Meeting, Thursday, February 23rd, 1899, at the Hospital.

National Orthopædic Hospital, 234, Great Portland Street, Regent's Park, W.—Annual Meeting, at four o'clock p.m., Tuesday, February 28th, 1899.

West Ham Hospital, Stratford, E.—Annual Meeting, at eight o'clock p.m., Wednesday, March 8th, 1899, in the Out-Patients' Hall of the Hospital.

London Fever Hospital, N.—Annual Meeting, February 10th, 1899, Hotel Victoria.

City of London Lying-in Hospital, City Road, E.C.—Annual Meeting, at three o'clock p.m., Wednesday, February 15th, 1889, at the Hospital.

Hospital for Consumption, Ventnor.—Annual Meeting, at four o'clock p.m., Thursday, February 23rd, 1899, at the Office, 34, Craven Street, Charing Cross.

Hostel of St. Luke.—Sixth Annual Meeting, at three o'clock p.m., Thursday, February 23rd, 1899, in the Church House, Dean's Yard, Westminster.

King's College Hospital.—Annual Meeting, at four o'clock p.m., Thursday, February 23rd, 1899, at the Hospital.

Charing Cross Hospital.—Annual General Meeting, at two o'clock p.m., February 22nd, 1899, at the Hospital.

Mr. James Mackenzie Davidson, M.B., C.M., has been appointed honorary medical officer in charge of the X-ray Department, Royal London Ophthalmic Hospital, Moorfields.

Dr. F. J. Waldo, medical officer of health of the Temple and Southwark, has been appointed Milroy lecturer by the Royal College of Physicians for the year 1900. The subject of the lectures will be: "Summer Diarrhœa, with Special Relation to Causation and Prevention."

Sir Douglas Powell delivered the first lecture to medical practitioners and to the students of the Hospital for Consumption and Diseases of the Chest, Brompton, on Wednesday last, the subject being Mitral Stenosis. Subsequent lectures and demonstrations of the course will be delivered on Wednesdays in February and March, at four p.m.

By direction of H.R.H. the Princess of Wales a cheque for £5 11s. 6d. has been received by the secretary of the British Home for Incurables, as the result of a further sale of Canon Fleming's sermon, "Recognition in Eternity," published by Messrs. Skeffington and Son. The British Home for Incurables was the first charitable institution in England to receive the honour of Her Royal Highness's patronage.

Further reports from Nice prove that the letter we published recently in our columns refuting the alarming rumours respecting typhoid fever in that place was correct. Although a slightly larger number of typhoid cases existed in the poorer part of the town, the epidemic did not spread at all amongst visitors and only two cases occurred amongst the domestics of permanent residents.

The evidence brought before the Plague Commission sitting at Lahore and Karachi is interesting, but not entirely convincing, owing to the different opinions formed by the various witnesses. One point, however, appears to meet with unanimous concurrence, and that is the efficacy of the evacuation of dwellings and their disinfection. The greatest divergence of opinion appears to be respecting the manner in which contagion is carried.

The dominant note in matters medical seems to be the provision for the treatment of consumption. Not only in England but on the Continent a general review of existing facilities is taking place. Germany is fairly provided with sanitoria, but in the Riviera, that resort of invalids, the absence of facilities for the pursuance of an outdoor life is naturally commented upon. The great variance of the temperature on the Riviera has been gradually recognised as a drawback where invalids are concerned, but with consumptives this would not be so deterrent as at present were sheltered accommodation in the open air provided for use after the dangerous sunset hour is past.

The final arrangements for the establishment of the Liverpool School for the Study of Tropical Diseases have now been made. The scheme has met with substantial local support. Mr. Jones' annual grant of £350 has been followed by promises from commercial circles connected with the port of about £900 per annum. The school will be connected with the Royal Southern Hospital and University College. A large ward has been set aside temporarily for the reception of patients suffering from tropical diseases. Connected with the ward is a good laboratory and convenient offices. The hospital annually receives a large number of patients from the docks suffering from the ailments of tropical climates.

The Distribution Committee of the Hospital Saturday Fund have advised, and it has been approved, that a sum of £17,030 16s. 5d. be awarded to the 173 participating institutions, viz., twenty-eight general hospitals, £6,238 7s.; six cottage hospitals, £260 10s.; sixty special hospitals, £5,131 1s.; thirty-two dispensaries, £879 2s.; twenty convalescent homes, £1,638 11s.; and twenty-seven miscellaneous institutions, £2,855 1s. Among the principal grants were: London Hospital, £879 5s.; Middlesex, £408 9s.; St. Mary's, £407 15s.; St. George's, £360 7s.; Guy's, £350; King's College, £348 6s.; University, £312 5s.; Seamen's, £309 7s.; Westminster, £293; Royal Free, £297 3s.; Charing, Cross, £287 8s.; West London, £195 16s.; Great Northern, £189 12s.; Metropolitan, £146; Temperance, £189; National for Epilepsy, £237 18s.; Royal London Ophthalmic, £183 8s.; Hospital for Women, Soho, £105 5s.; Cancer (Brompton), £405 6s.; City of London (Consumption), £242 17s.; Hospital for Sick Children, £310 16s.; East London, for Children £210 19s.; Victoria, for Children, £208 5s. The total receipts for 1898 were £19,338 8s. 4d. (a decrease of £669 upon the previous year). The awards were £500 less than last year.

